# Optimizing Antimicrobial Therapy by Integrating Multi-Omics With Pharmacokinetic/Pharmacodynamic Models and Precision Dosing

**DOI:** 10.3389/fphar.2022.915355

**Published:** 2022-06-23

**Authors:** Hui-Yin Yow, Kayatri Govindaraju, Audrey Huili Lim, Nusaibah Abdul Rahim

**Affiliations:** ^1^ Faculty of Health and Medical Sciences, School of Pharmacy, Taylor’s University, Subang Jaya, Malaysia; ^2^ Centre for Drug Discovery and Molecular Pharmacology, Faculty of Health and Medical Sciences, Taylor’s University, Subang Jaya, Malaysia; ^3^ Department of Pharmaceutical Life Sciences, Faculty of Pharmacy, Universiti Malaya, Kuala Lumpur, Malaysia; ^4^ Centre for Clinical Outcome Research (CCORE), Institute for Clinical Research, National Institutes of Health, Shah Alam, Malaysia; ^5^ Department of Clinical Pharmacy and Pharmacy Practice, Faculty of Pharmacy, Universiti Malaya, Kuala Lumpur, Malaysia

**Keywords:** antimicrobial therapy, pharmacokinetic/pharmacodynamic (PK/PD), mechanism-based PK/PD models, multi-omics, systems pharmacology, precision dosing

## Abstract

In the era of “*Bad Bugs, No Drugs*,” optimizing antibiotic therapy against multi-drug resistant (MDR) pathogens is crucial. Mathematical modelling has been employed to further optimize dosing regimens. These models include mechanism-based PK/PD models, systems-based models, quantitative systems pharmacology (QSP) and population PK models. Quantitative systems pharmacology has significant potential in precision antimicrobial chemotherapy in the clinic. Population PK models have been employed in model-informed precision dosing (MIPD). Several antibiotics require close monitoring and dose adjustments in order to ensure optimal outcomes in patients with infectious diseases. Success or failure of antibiotic therapy is dependent on the patient, antibiotic and bacterium. For some drugs, treatment responses vary greatly between individuals due to genotype and disease characteristics. Thus, for these drugs, tailored dosing is required for successful therapy. With antibiotics, inappropriate dosing such as insufficient dosing may put patients at risk of therapeutic failure which could lead to mortality. Conversely, doses that are too high could lead to toxicities. Hence, precision dosing which customizes doses to individual patients is crucial for antibiotics especially those with a narrow therapeutic index. In this review, we discuss the various strategies in optimizing antimicrobial therapy to address the challenges in the management of infectious diseases and delivering personalized therapy.

## Introduction

Antibiotics have been regarded as one of the most instrumental advances in modern healthcare from the beginning of their discovery until the present day in controlling infectious diseases that were the leading causes of human morbidity and mortality. However, despite their indispensable contribution to global healthcare, their equilibrium in the arms race against microorganisms is fragile. Inappropriate and overuse of antibiotics lead to the emergence and spread of antibiotic-resistant bacteria in the community, which significantly threaten human health and the global economy. In 2019 the World Health Organization (WHO), reported that antimicrobial resistance (AMR) is one of the top 10 global public health threats facing humanity. The acceleration of antibiotic resistance is one of the most alarming consequences of antibiotic overuse. According to a recent study, 50% of all the antibiotics prescribed for people are not needed or are not optimally effective as prescribed in US hospitals (Magill et al., 2021).

AMR occurs naturally over time, usually through genetic changes, and antibiotics are becoming increasingly ineffective as drug resistance spreads globally, making it more difficult to treat infections and death. At the heart of this problem is the dearth of antimicrobial drugs development in the clinical pipeline. In 2019, WHO identified 32 antibiotics in clinical development that address the WHO list of priority pathogens, of which only six were classified as innovative. The findings clearly highlight the pressing need for greater innovation and investment in developing new antimicrobials for efficient control and management of infectious diseases.

Presently, the progress in the development of antimicrobials is driven by the modification of existing classes of antimicrobials rather than by the discovery of new antimicrobial classes (Aminov, 2017). Thus, hindering the discovery of new classes of antimicrobials for decades. However, acknowledging the battle against rapidly emerging bacterial resistance is a relentless clinical problem and cannot be solved once and for all; we can no longer rely entirely on discovering new antibiotics. Instead, implementing better strategies for the use of older and readily available antibiotics would be worthwhile pursuing to handle the problem as efficiently and safely as possible. These strategies should be formulated based on how antimicrobial resistance develops and on identifying critical checkpoints where preventive measures could be imposed to stop or at least hamper the process (Chernov et al., 2019).

One of the strategies to contain the rapid expansion of resistance could be to emphasize the reengineering and optimization of existing antimicrobials since the new antimicrobial drug development has been largely focused on extensive modifications of existing natural drugs. For example, due to toxicity concerns, antibiotics such as chloramphenicol which may cause neurotoxicity, and haematological disorders have had derivatives developed, including florfenicol and thiamphenicol, which exhibit less toxicity (Dinos et al., 2016). However, to date, florfenicol and thiamphenicol have only been used in animals (Shin et al., 2005; Wei et al., 2016). Promisingly, the application of pharmacokinetic and pharmacodynamics (PK/PD) strategies may allow more therapeutically effective use of some existing antibiotics. Antimicrobials that have previously been shelved due to toxicity concerns, such as daptomycin and colistin, are now being used to treat life-threatening infections, highlighting the importance of PK/PD data in the optimal use of old antimicrobials (Ortwine et al., 2015). Another strategy worthy of consideration is combining antibiotics with non-antimicrobial compounds that display synergistic effects to extend the useful life of some older antibiotics (Ejim et al., 2011). Mechanism-based PK/PD models have also been developed to further investigate and inform optimal dosing regimens. Moreover, the use of omics technologies could substantially contribute to the discovery/development of these compounds and identify novel targets. The advances in transcriptomics, proteomics, and metabolomics permit the profiling of bacteria during antimicrobial exposure and have revealed the involvement of many pathways in antimicrobial response and resistance (Pulido et al., 2016). This has led to the emergence of quantitative systems pharmacology (QSP) and model-informed precision dosing (MIPD).

Herein in this review, we discuss the various strategies for optimizing antimicrobial therapy to address the challenges in the management of infectious diseases and delivering personalized therapy.

## PK/PD Considerations When Optimizing Antibiotic Dosing

**FIGURE 1:**
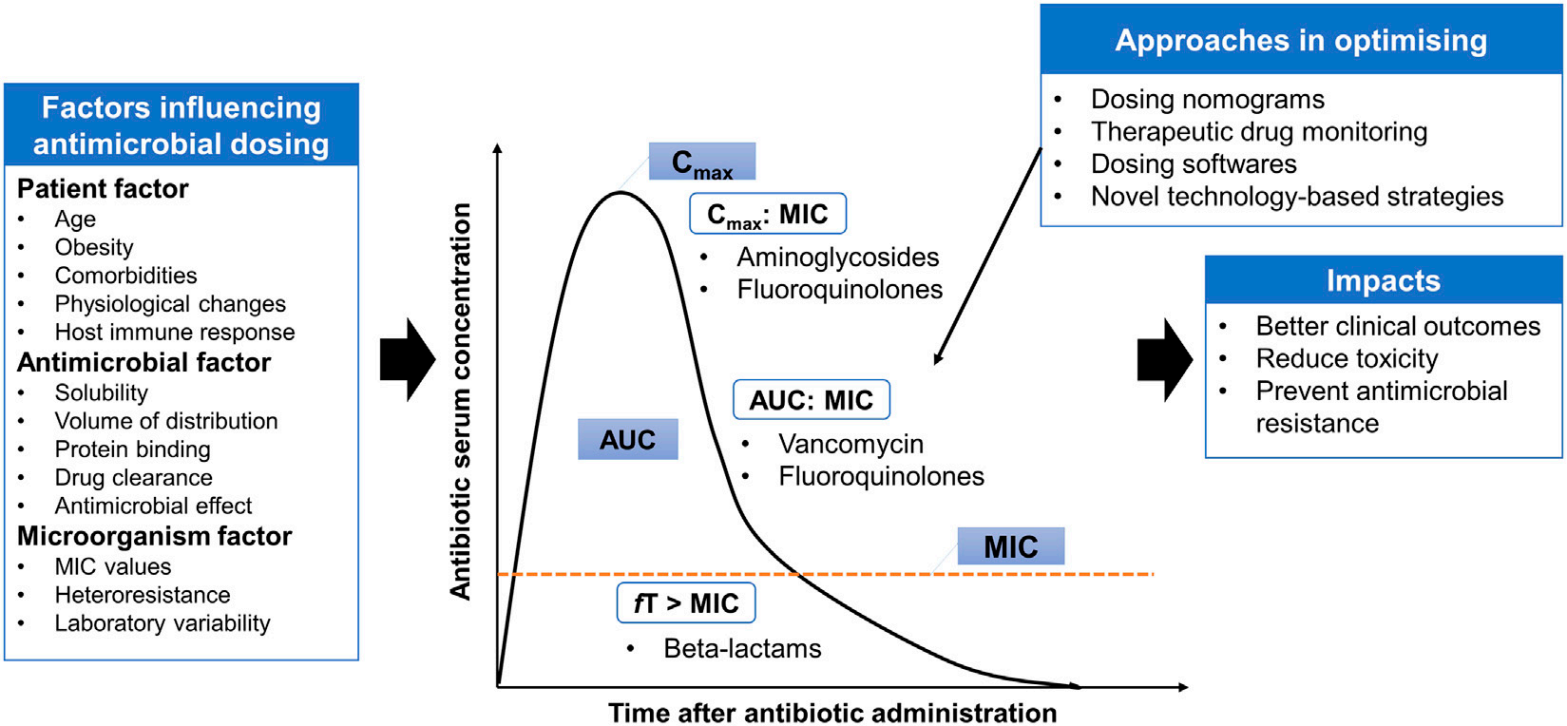
Antimicrobial pharmacokinetic-pharmacodynamic is affected by three main factors: patient, antimicrobial and microorganism factors. Several approaches are implemented in the clinical practice settings or under the experimental phase to provide individualized dosing and address some degree of variabilities. AUC: area under the curve; MIC: minimum inhibitory concentration; C_max_: maximum concentration; *f*T > MIC: Time that free serum concentration above minimum inhibitory concentration; C_max_:MIC: ratio of maximum concentration to minimum inhibitory concentration; AUC:MIC: ratio of area under the concentration-curve to minimum inhibitory concentration.

In addition to the appropriateness of antimicrobial agent selection (both mono- and combination therapies), optimal antimicrobial dosing is another key factor for therapeutic success in managing infectious diseases, while minimizing the toxicity and preventing the emergence of antimicrobial resistance (Figure 1). Suboptimal antimicrobial dosing has been associated with poorer clinical outcomes, in terms of clinical cure rate and mortality (Roberts et al., 2014; Appaneal et al., 2021). This is attributed to inadequate drug exposure in achieving PK/PD targets in individual patients (Hoo et al., 2017). This issue is closely related to the physiological changes of patients, which are commonly observed in critically ill, comorbidities, elderly and obese patients (Pai and Bearden, 2007; Chai et al., 2020; Rawson et al., 2021). Pharmacokinetic variation is well-explained in critically ill patients, who often present with altered pharmacokinetic parameters. These include fluid shifts due to capillary leak syndrome leading to an increase in the volume of distribution of hydrophilic antimicrobial agents (e.g., beta-lactams and aminoglycosides) and a decrease in plasma concentration, hypoalbuminemia causing changes in protein binding for high protein-bound antimicrobials (e.g., ertapenem and flucloxacillin) and organ dysfunction that reduces or increases the drug clearance through renal and hepatic systems and therefore leading to pharmacokinetic variability (Cai et al., 2012; Chai et al., 2020; Rawson et al., 2021).

Besides inter-individual variability, antimicrobial dosing is also affected by two other factors: antimicrobial and microorganism factors. Antibiotics available vary in physicochemical and pharmacokinetic properties, including solubility, the volume of distribution, protein binding and drug clearance (Hoo et al., 2017). These properties must be taken into consideration to estimate the antimicrobial disposition in the body. To understand the application of antimicrobial dosing to efficacy, the pharmacology of antimicrobial agents in terms of pharmacokinetic and pharmacodynamic profiles need to be integrated (Table 1). Antimicrobial dosing is conventionally prescribed using a fixed-dose based on a one-size-fits-all approach, where pharmacokinetics and pharmacodynamic variabilities are not taken into account and the antimicrobial dosing regimen is chosen according to the drug exposure and pharmacokinetic data from the general population (Tuntland et al., 2014). Together with the pharmacokinetic and pharmacodynamic variabilities driven by patients, determining optimal dosing of antimicrobial agents for individual patients is challenging.

**TABLE 1:** Pharmacokinetic characteristics of commonly used antibiotics with their pattern of killing and pharmacokinetic/pharmacodynamic target.

Antibiotic	Pharmacokinetic properties (Hoo et al., 2017)				Pattern of antimicrobial activity	PK/PD index (Kowalska-Krochmal & Dudek-Wicher 2021; Rawson et al., 2021)
	Solubility	V_d_ ^a^	Protein binding	CL		
Beta-lactams	Hydrophilic	Low	Low to moderate^b^	Renal	Time-dependent	*f*T > MIC
Vancomycin	Hydrophilic	Low	Moderate	Renal	Time- and concentration dependent	AUC:MIC
Fluoroquinolones	Lipophilic	Moderate	Low to moderate	Hepatic and renal	Concentration-dependent	C_max_:MIC AUC:MIC
Aminoglycosides	Hydrophilic	Low	Low	Renal	Concentration-dependent	C_max_:MIC

a Low V_d_: 0.1–0.4 L/kg, moderate V_d_: 0.6–5 L/kg.

b Exceptions: Cefazolin (75%–85%), ceftriaxone (85%–95%), ertapenem (85%–95%), flucloxacillin (95%), dicloxacillin (97%), oxacillin (94%). V_d_, volume of distribution; CL, clearance; fT > MIC, time that free serum concentration above minimum inhibitory concentration; C_max_:MIC, ratio of maximum concentration to minimum inhibitory concentration; AUC:MIC, ratio of area under the concentration-curve to minimum inhibitory concentration.

Unfortunately, antimicrobial dosing is further complicated by the susceptibility of the pathogen to the antimicrobial agent, which is determined by measuring the minimum inhibitory concentration (MIC) of the antimicrobial agent in inhibiting microbial growth. MIC is the major indicator of antimicrobial effectiveness (Kowalska-Krochmal and Dudek-Wicher 2021). It is the denominator in the PK/PD index, which describes the quantitative relationship between the given dose and the bacterial killing effect in terms of rate and extent of killing (Hoo et al., 2017). European Committee on Antimicrobial Susceptibility Testing (EUCAST) and Clinical and Laboratory Standards Institute (CLSI) classified the susceptibility breakpoints that are useful for dose optimization depending on MIC values, which is vital when local laboratory antibiograms are not available (Clinical & Laboratory Standards Institute, 2022; European Committee on Antimicrobial Susceptibility Testing, 2022). The accuracy in MIC determination is subjected to a few factors, such as variation in a laboratory (intra-laboratory variability) and variation in determination method (intra-sample variability) (Mouton et al., 2018; Kowalska-Krochmal and Dudek-Wicher 2021). On the other hand, several studies reported that pathogens isolated from critically ill patients were less susceptible to antimicrobial agents with the presence of higher MIC values (Kiratisin et al., 2012; Leblebicioglu et al., 2012; Valenza et al., 2012; Pérez-Pitarch et al., 2018). This indicates that higher antimicrobial dosing is needed to achieve the PK/PD targets for better clinical outcomes.

In order to achieve optimal antimicrobial dosing, several approaches are implemented in the clinical practice settings or under the experimental phase to provide individualized dosing and address some degree of variabilities. These include dosing nomograms, therapeutic drug monitoring (TDM), dosing software and other novel technology-based approaches, such as real-time drug monitoring using biosensors, closed-loop controlled systems, artificial intelligence and machine learning assisted systems (He et al., 2021; Rawson et al., 2021). These technology-based approaches have several barriers to applying in clinical practice including inadequate investigation on their applications, the issue of data integration with patient medical records (Rawson et al., 2021). Thus, advanced strategies that can offer rapid and precise antimicrobial dosing adjustments are needed to optimize the antimicrobial dosing. TDM and model-informed precision dosing (MIPD), which is an emerging approach, are further addressed in the sections below.

## Pharmacology and Mechanism-Based PK/PD Models

It is well known that bacteria behave differently *in vitro* and *in vivo*. As such, in antibiotic discovery and development, PK/PD studies are imperative to provide substantial insight into the therapeutic potential of lead compounds at the early discovery stage and assist in the establishment of optimal dosage regimens (de Araujo et al., 2011; Velkov et al., 2013). As Schmidt et al. (2008) described, ideal treatment optimization requires information on the mechanisms involved in the effect of the antibiotics (pharmacodynamics, PD) and the evolution of the antibiotic concentration in the patients (pharmacokinetics, PK).

PK is a central part of clinical pharmacology and pharmacometrics. It describes the relationship between drug dosing and the drug concentration-time profile in the body. Whilst PD describes the relationship between the concentration of an antibiotic and its ability to inhibit the growth of microorganisms. The major pharmacodynamic parameter is the drug’s minimum inhibitory concentration (MIC) against the infecting pathogen. Despite MIC’s well-established susceptibility parameter, which has been paramount to understanding antimicrobial dosing, it is a crude and mono-dimensional threshold value. The value can show high variability and does not provide information on the time course of antimicrobial activity or growth inhibition due to antibiotic exposure (Craig, 2003). In comparison, the evaluation of growth and kill profiles over time (time-kill curves) offer a more robust approach.

PK/PD models link the dose/concentration relationship (PK) and concentration/effect relationship (PD), thereby facilitating the description and prediction of the clinically relevant relationship between time and drug effects (Schmidt et al., 2008). The PK/PD approach implies the use of *in vitro*, *ex vivo*, and *in vivo* models, as well as mathematical models (Rodríguez-Gascón et al., 2021). Each one exhibits advantages and disadvantages and may be regarded as complementary. The mathematical modelling to analyze PK/PD data resulting from *in vitro*, *ex vivo* or *in vivo* experiments has an important impact on the development and optimization of antibiotic dosing.

Besides its main application, to optimize dosing strategies to improve the clinical outcome of antibiotic therapy, the PK/PD analysis also minimizes side effects and the emergence of resistances (Asín-Prieto et al., 2015). Furthermore, the PK/PD indices define the combination of pharmacokinetic and pharmacodynamic parameters. For instance, the ratio of the peak concentration of the antimicrobial (C_max_) to the minimum inhibitory concentrations (MICs) (C_max_/MIC) (McAleenan et al., 2020).

Three PK/PD indices have been set as the best descriptors of clinical efficacy and bacterial kill characteristics of the antibiotic based on the activity pattern of the antibiotic (Table 1). The first pattern of antimicrobial activity exhibits concentration-dependent activity and the PK/PD indices preferred are the ratios of the free-drug maximum concentration (*f*C_max_) to the MIC (*f*C_max_/MIC) or the area under the free-drug concentration-time curve, typically over a 24-h period, to the MIC (*f*AUC_24_/MIC). The second is the time-dependent pattern, where the antibacterial effect is best described by the percentage of time the free drug concentration remains above the MIC throughout the dosing interval (*f*T_>MIC_). Finally, the best PK/PD ratio for concentration-dependent with time-dependence antibiotics is *f*AUC_24_/MIC (Jorda and Zeitlinger, 2020; McAleenan et al., 2020).

Over the last decades, the regulatory agencies recommended model-based drug development to strengthen scientific evidence as a basis for making key decisions. However, very few PK/PD models describe time courses of antibiotic drug effects in animals and patients. The model-based has its drawbacks as only a few PK/PD models describe time courses of antibiotic drug effects in animals and patients. To overcome the drawback, the mechanism-based model could help predict the time-course of bacteria growth and kill in patients, based on *in vitro* and/or *in vivo* information compared with more empirical models. Notably the mechanism-based PK/PD model (MBM) are more reliable for extrapolating different dosing regimens in the presence of resistant mutants in investigating how resistance selection can be reduced or overcome (Danhof et al., 2008; Khan et al., 2015).

The MBM includes equations that describe microorganism growth, the effect of antimicrobial drugs, and changing drug concentrations (Czock and Keller, 2007). The MBM takes into account several parameters, including MIC value, at minimum, a control growth rate constant (K_growth_) and a killing rate (K_death_), a maximum kill rate (E_max_) and a potency value such as the half-maximum effect concentration (EC_50_) (Nielsen and Friberg, 2013). Moreover, as only limited information on the drug effect may be needed when the underlying system is characterized, MBM can be useful for selecting between candidate drugs. As such, it allows more therapeutically effective use and has renewed interest in some old antibiotics. For example, colistin is administered as a last-line therapy for difficult-to-treat respiratory tract infections via intravenous administration or inhalation (Lin et al., 2018). However, both routes fail to achieve adequate exposure owing to poor penetration into the epithelial lining fluid and a lack of scientific evaluation with well-designed PK/PD studies, respectively (Landersdorfer et al., 2017; Yapa et al., 2014). When an MBM was used, optimal inhalational dosage regimens of colistin were developed to treat life-threatening respiratory tract infections caused by Gram-negative superbugs in patients (Lin et al., 2018). Nielsen et al. (2007) have previously developed a mechanism-based *in silico* model that successfully described the bacterial growth and killing kinetics for *Streptococcus pyogenes* exposed to antibiotics of different classes. The Nelson model structure provides valuable information for future studies’ design and the development of improved dosing regimens. Therefore, the efforts taken to develop this model are rewarding in the battle against rapidly emerging bacterial resistance as we can no longer rely entirely on discovering new antibiotics.

Following established MBMs using parameter estimates including bacterial growth, bacterial killing and mutation frequency with the application of relevant software, dosing regimens of antibiotics can be further optimized *via* Monte Carlo simulation in conjunction with human population PK models. Monte Carlo simulation is a mathematical technique which can be performed to determine the probability of target attainment (PTA) under different dosing regimens by utilizing PK data and PK/PD targets (Llanos-Paez et al., 2017; Trang et al., 2017). The Monte Carlo simulation combines PK and microbiological data to predict the therapeutic outcome for different antimicrobial dosage regimens. Thus acting as an additional tool that can support antimicrobial dose optimization and guide empiric therapy. Overall, these *in silico* PK/PD models have been essential in optimizing therapy, including antibiotics dosage regimens (Yadav et al., 2017).

## Multi-Omics and Metabolic Modelling

**FIGURE 2:**
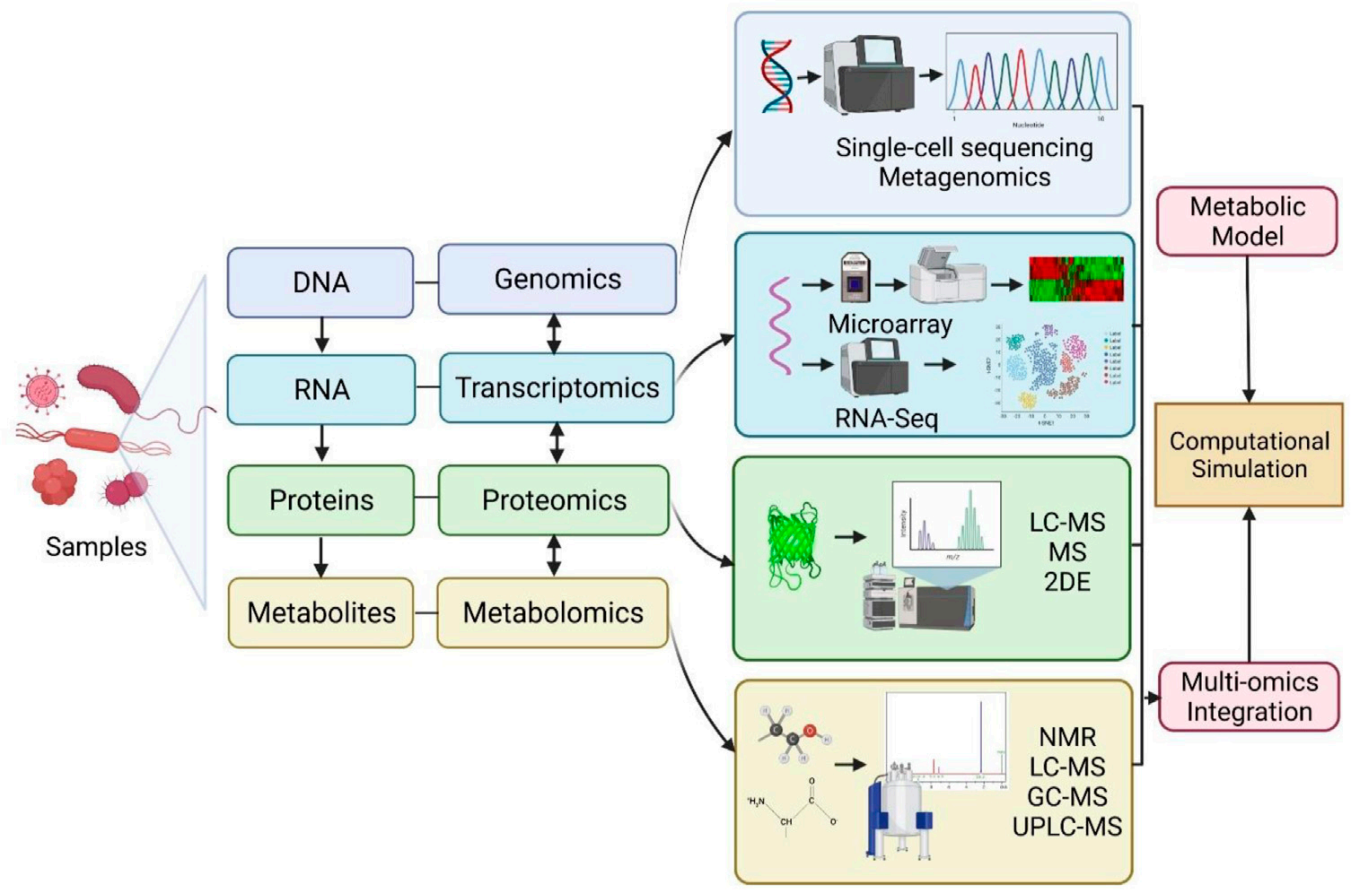
Schematic representation of omics workflow for genomics, transcriptomics, proteomics and metabolomics approaches. Multi-omics data integration can be used for refining and reconciling modelling predictions to construct computational simulations. RNA-Seq: RNA-sequencing; LC-MS: liquid chromatography-mass spectrometry; MS: Mass spectrometry; 2DE: two-dimensional electrophoresis; NMR: nuclear magnetic resonance; GC-MS: gas chromatography coupled to mass spectrometry; UPLC-MS: ultra-high-performance liquid chromatography coupled to mass spectrometry. Created with BioRender.com.

Conventionally, growth inhibition assays in disk-diffusion, well diffusion, broth or agar dilution can be used for epidemiology, drug discovery and prediction of therapeutic outcomes (Balouiri et al., 2016). However, the procedures are laborious and time-consuming as only a few isolates can be studied at one time, while some methods are highly subjected to the risk of errors in the preparation of dilutions or determining MIC values (Balouiri et al., 2016). Recent advances in “omics,” which is an umbrella term for genomics, transcriptomics, proteomics, lipidomics and metabolomics, are emerging and provide valuable tools in getting deeper insights into bacterial physiology and virulence mechanism of antimicrobial resistance and mechanisms of potential antimicrobial compounds (Table 2). The schematic representation of omics workflow is depicted in Figure 2. The integration of high-throughput multi-omics data can unravel the relationship between genes and proteins, as well as the interaction of biological networks in a system-based model (Garcia et al., 2021). This explains the reason for the growing interest in using multi-omics analysis in microbiology research.

**TABLE 2:** Examples of microbial studies used omics technologies.

Omics strategies	Approach	Study objective	Drug/ compound	Pathogen	Reference
Genomics	Single-cell sequencing	Evaluate human microbiota	—	Microbiota of a healthy oral subject	Campbell et al. (2013)
Genomics	Single-cell sequencing	Identify bacteria that affect disease susceptibility and severity	—	Intestinal microbiome from 11 patients with inflammatory bowel disease	Palm et al. (2014)
Genomics and metagenomics	Single-cell sequencing + Shotgun sequencing	Evaluate the genomes of SAR86 marine bacterial lineage	—	SAR86 from seawater	Dupont et al. (2012)
Metagenomics	Shotgun sequencing	Assess health risk of antimicrobial resistance genes (ARGs)	—	1,921 gut microbiome genomes from 59 healthy stool donors	Zhang et al. (2021)
Metagenomics	Shotgun sequencing	Investigate the rates and targets of horizontal gene transfer (HGT) across thousands of bacterial strains	—	Samples were collected from 15 human populations spanning a range of industrialization	Groussin et al. (2021)
Transcriptomics	RNA-Seq	Analyze the regulation of adaptive resistance upon adaptation to disparate toxins	Ampicillin, tetracycline, n-butanol	*E. coli*	Erickson et al. (2017)
Transcriptomics	Microarray	Identify molecular mechanism of Licochalcone A	Licochalcone A from *Glycyrrhiza inflata*	*S. aureus*	Shen et al. (2015)
Transcriptomics, metabolomics, lipidomics and lipid A profiling data	Genome-scale metabolic modelling	Analyze bacterial metabolic changes at the systems levels	Polymyxins	*P. aeruginosa*	Zhu et al. (2018)
Proteomics	nanoLC-MS/MS	Analyze bacterial phosphoproteomic changes of prokaryotes for drug resistance	-	*A. baumannii, H. pylori, K. pneumoniae, V. vulnificus, A. platensis, M. taiwanensis, T. thermophilus, M. mazei, M. portucalensis*	Lai et al. (2017)
Proteomics	MS and 2D-DIGE	Identify changes in subproteome	Piperacillin/ tazobactam	*E. coli*	dos Santos et al. (2010)
Proteomics	2DE and iTRAQ	Investigate the mechanism of Plumbagin	Plumbagin	*B. subtilis*	Reddy et al. (2015)
Metabolomics and proteomics	Computational model	Identify the biomarkers to predict patient outcomes and guide therapeutic development	-	*S. aureus*	Wozniak et al. (2020)
Metabolomics	HPLC with MS	identify metabolic changes of bacteria	Methicillin, ampicillin, kanamycin, norfloxacin	Two isogenic *S. aureus* strains	Schelli et al. (2017)

Nano LC-MS/MS, nanoscale liquid chromatography coupled to tandem mass spectrometry; MS, mass spectrometry; 2D-DIGE, two-dimensional difference gel electrophoresis; 2DE, two-dimensional electrophoresis; iTRAQ, isobaric tag for relative and absolute quantification; HPLC, high performance liquid chromatography.

The advent of genetic sequencing technologies over the past decade, together with the bioinformatics tools, enable the decoding of microbial genomes with better taxonomic and functional annotation of genomic and metagenomic data, which leads to the identification of novel genes related to bacterial virulence, resistance and mutations (Roemer & Boone 2013; Scheffler et al., 2013). The incorporation of genomic technologies into microbiology research provides a large genomic dataset which is valuable in predicting the metabolic pathway of microorganisms (Garza and Dutilh, 2015), identification of promising candidate targets for novel antimicrobials in drug discovery and development (Santos et al., 2016), elucidating the antimicrobial resistance mechanisms via comparative genomic analysis (Chernov et al., 2019). Single-cell sequencing and metagenomic sequencing are widely used approaches for investigating microbiome, which refers to the microorganisms inhabiting a particular environment (Cheng et al., 2019). Single-cell sequencing provides high-quality genomic information for microbe strains with low abundance (which might be missing from metagenomic sequencing), by isolating individual cells for DNA extraction, whole-genomic amplification, high-throughput sequencing and lastly followed by genome assembly and genome analysis (Cheng et al., 2019). The application of single-cell sequencing in microbiology research has led to significant findings, including the discovery of bacteria with novel metabolic features and alternative genetic code (Campbell et al., 2013), and the identification of microbial taxa that induce specific disease (Palm et al., 2014).

However, single-cells genomics represents genomes of individual cells and may not provide the full genomic collection within a microbial community or biome. This shortcoming can be solved with the integration of metagenomics and single-cell genomics (Cheng et al., 2019). Metagenomics allows the assembly of basic genomes with microbial communities by investigating the whole genomes of all microbes in microbial communities contained within a certain environment. Antibiotic resistance can be transferred from one bacterium to another *via* horizontal gene transfer. A recent report demonstrated that it occurs at an elevated rate in the gut microbiome among individuals from industrialized and urban populations (Groussin et al., 2021). This approach has been employed to investigate microbial diversity and antibiotic resistance genes (Dupont et al., 2012; Zhang et al., 2021). Integration of single-cell genomics and metagenomics produced better microbe genome assemblies from microbial communities (Dupont et al., 2012; Nobu et al., 2015).

Transcriptomics involves large-scale analysis of transcriptional changes in gene expression levels, which are produced by the microbes in response to a defined environmental condition, such as antimicrobial treatment. The mechanism controlling the activity of antimicrobial agents on microbial cells involves a complex interaction of multiple pathways at different levels, such as transcriptional, translational and post-translational levels (Dwyer et al., 2015; Erickson et al., 2017). Gene expression analysis, together with bioinformatics resources, may help in unveiling the mechanism of antimicrobial action (Shen et al., 2015), metabolic changes of bacteria (Zhu et al., 2018) and mechanism of bacterial adaptive resistance (Erickson et al., 2017). The microarray approach using microchip technology allows simultaneous analysis on gene expression levels in microbes at the transcription level, where the gene expression patterns (up-regulation or down-regulation) of microbes in response to a stimulus can be identified. Therefore, it can provide insights into the molecular mechanisms or pathways involved in the investigated phenomena at a systems level. It has been applied in microbiology research to elucidate the mechanism of action of several potential antimicrobial agents (Shen et al., 2015; Santos et al., 2016).

The application of microarray or gene probe-based methods are limited to the availability of the array for the specific microbe strain, completeness of genome sequence of an investigated organism, issue of result reproducibility and availability of instrument to read microchip (Chernov et al., 2019). As an alternative, the RNA sequencing (also termed as RNA-Seq) method is employed to provide quantitative analysis of gene expression, and at the same time, it allows the gene profiling of non-coding RNAs involved in regulating gene expression at transcriptional and post-transcriptional levels. Unlike probe-based approaches, RNA-Seq can be used to detect the transcripts in an organism without a complete sequenced genome and provides high accuracy and reproducibility in quantifying gene expression levels (Wang et al., 2009).

The assessment of protein expression level (qualitatively or quantitatively) at the whole-cell level can be achieved *via* proteomics analysis. It allows protein identification and evaluation of differential protein expression for the investigated phenomena, as well as provides information on post-translational modifications of proteins (Chernov et al., 2019). This information highlights the key proteins involved, pathways affected and the underlying mechanisms in response to stimuli, including identification of bacteria proteome and mechanism of drug resistance (Lai et al., 2017; dos Santos et al., 2010). Proteomic approaches are also adopted in drug discovery research to investigate the mechanism of actions of potential antimicrobial agents and reveal their cellular targets (Reddy et al., 2015). Various methods can be used for proteomics analysis, such as mass spectrometry and two-dimensional electrophoresis, liquid chromatography-mass spectrometry (LC-MS) (Lai et al., 2017; dos Santos et al., 2010).

Metabolomics offers the metabolic profiling of an organism by determining the metabolites present over a given time under certain conditions. In other words, it provides a snapshot of cell physiology in view of metabolites as end-products of cellular processes. Similar to proteomics, various approaches can be used for metabolomics, such as nuclear magnetic resonance (NMR), LC-MS, gas chromatography coupled to mass spectrometry (GC-MS) and ultra-high performance liquid chromatography coupled to mass spectrometry (UPLC-MS) (Chernov et al., 2019). This technology may help in determining metabolic changes related to antimicrobial resistance (Schelli et al., 2017), disease, toxins or drugs (Santos et al., 2016).

To unveil the complexity of the microbial system, data from multi-omics are integrated into metabolic modelling to improve model predictions and provide comprehensive insights into cellular networks at the system level (Fondi and Liò 2015). This is due to the limitation of inaccuracy prediction in a single layer network. Genomic-scale cellular network has become an important tool to bridge the gap between genomic-derived data (such as gene products, mRNA, proteins and metabolites) and their interactions, offering a comprehensive understanding of the dynamic physiological function of microbes under a specific condition (Hao et al., 2018). The cellular networks have been classified into: 1) genomic-scale metabolic network—allows systematic level predictions of metabolism in the organism, 2) transcriptional regulatory network—allows predictions of interactions between different transcriptional factors and target genes, and 3) signal transduction network—allows predictions of molecular cell response (protein-protein ad protein-gene interactions) to stimuli (Hao et al., 2018). Zhu et al. (2018) integrated multi-omics data with a genome-scale metabolic model (GSMM) to analyze the responses of *P. aeruginosa* to polymyxins. Abdul Rahim et al. (2021) employed a systems-based model to analyze the synergistic activity of polymyxin B and chloramphenicol. Wozniak et al. (2020) adopted a combination of metabolomics and proteomics analysis of *S. aureus* bacteraemia together with the computational tool as biomarkers to predict mortality risk and recommend personalized therapy.

In addition to exploring the genome information of the microbes and the response of the antimicrobial agents, multi-omics also provide a meaningful approach to address the inter-individual variability through exploration of genomic information from the host, which is important in optimizing antimicrobial therapy. Genetic polymorphisms of drug-metabolizing enzymes were reported to be associated with anti-tuberculosis drug-induced hepatitis, including NAT2*4 and CYP2E1*4 (Kim et al., 2009; Cai et al., 2012). These pharmacogenomic data are important in predicting the clinical response to antimicrobial agents and tailor an individualized antimicrobial therapy. Interestingly, mitochondrial pharmacogenomics is also useful to provide a personalized barcode for antimicrobial therapy in view of the mutational rate of mitochondrial DNA is much higher than nuclear DNA (Pacheu-Grau et al., 2010; Singh et al., 2014). Mitochondrial DNA genetic variants were found to be associated with different aminoglycosides-related ototoxicity susceptibility (Rydzanicz et al., 2010; Muyderman et al., 2012). Integrating the data from pharmacogenomics into the drug’s PK/PD could provide an approach in optimizing antibiotic doses while minimizing the toxicity of antimicrobial agents.

On the other hand, multi-omics technology is also supported by European Union as a framework for developing an omics-based personalized treatment scheme as an approach in addressing the emergence of antibiotic resistance (Cohen et al., 2015). This tailored-treatment approach integrates personalized patient data such as identification of genetic predisposition to specific infections (host genomics), host immune response to infections (proteomics, transcriptomics), microbiome analysis (transcriptomics) and characterization of pathogen resistance profile (microbial genomics) to generate a database and this database will be mined using bioinformatics tools to identify the significant associations between different datasets. This eventually will contribute to the building of treatment algorithms to implement the personalized treatment with an optimal treatment regimen for individual patients (Cohen et al., 2015).

## Quantitative Systems Pharmacology

As highlighted above, PK/PD consideration is essential for antimicrobial dose optimization. Moreover, with multi-omics, there has been a paradigm shift from MBM to systems-based models. In recent years, there has been an increase in interest in quantitative systems pharmacology (QSP) (Ribba et al., 2017). QSP is an innovative, emerging interdisciplinary approach that integrates systems biology and PK/PD (Pichardo-Almarza and Diaz-Zuccarini, 2016). QSP modelling technique applies biology with findings of *in vitro* studies to determine the way drugs affect biological processes (Woodhead et al., 2017). It differs from other pharmacometrics approaches as QSP modelling enables the elucidation of the mechanisms of action and possibly resistance of drugs upon exposure on a systems level. This is done by quantitatively analyzing the dynamic interactions between drug(s) and a biological system (van der Graaf & Benson 2011). Unlike the traditional mechanism-based PK/PD models, which apply minimal mechanistic biology, QSP incorporates omics data, including transcriptomics and metabolomics, along with computational techniques to incorporate key interactions between the drug and its targets which result in changes in cellular processes (Zhao & Iyengar 2012).

This promising approach has led to the release of a US National Institutes of Health (NIH) white paper in 2011 (Sorger et al., 2011). There are many different applications of QSP, including evaluating the impact on the efficacy of novel mono- and combination therapies (Musante et al., 2017). The application of QSP modelling is evident in neoplasms, nervous system, cardiovascular system and nutritional and metabolic disease (Knight-Schrijver et al., 2016). Nonetheless, there are still barriers to QSP. Among them is the complexity of the models, and hence simplification via model reduction is recommended (Derbalah et al., 2022). Others include the cost and time of model development due to the large amount of datasets (Garcia et al., 2021).

To date, no QSP models have been published for antibiotics against “superbugs.” Due to the rapidly emerging resistance of MDR Gram-negative bacteria, these *in silico* models are urgently needed to optimize antibiotic combinations and hence to meet the needs of the current global health problem.

## Model-Informed Precision Dosing

Another emerging approach is model-informed precision dosing (MIPD). Treatment response in individuals for some drugs may vary due to genotype and phenotype differences (Peck, 2021). In order to ensure optimal outcomes in patients, drugs with certain characteristics would benefit from close monitoring and precision dosing (i.e., dosing tailored to individual patients). These characteristics include drugs with a narrow therapeutic index which may lead to serious adverse effects from overtreatment or severe consequences due to suboptimal treatment (Peck, 2021). Pharmacokinetics is the study of drug concentration changes in the body that results from various physiological processes over time. To identify significant covariates of response variability, population pharmacokinetic (popPK) modelling can be utilized for dose adjustments. The approach of using Bayesian popPK modelling to optimize dosing is called model-informed precision dosing (MIPD) (Abdulla et al., 2021). As PK/PD is employed, MIPD can be carried out if drug plasma concentrations are useful in predicting pharmacological effects.

Antibiotics with a narrow therapeutic window require close monitoring and are done with a therapeutic drug monitoring service (TDM). TDM service is offered in hospitals globally and is usually led by pharmacists (Ab Rahman et al., 2013). TDM involves the interpretation of drug plasma concentration levels which are compared to a therapeutic range. Based on this, recommendations are made, which often involve dose adjustments in order to optimize outcomes. Examples of antibiotics where TDM is conducted are vancomycin and aminoglycosides (amikacin and gentamicin). Suboptimal doses may lead to treatment failure and the emergence of AMR. Increased exposure, often demonstrated by levels above the recommended therapeutic range for vancomycin, amikacin and gentamicin, would lead to nephrotoxicity (Morales-Alvarez, 2020). Nonetheless, serious adverse effects and toxicities may also develop in patients with plasma concentration levels well within the therapeutic range.

Although TDM can assist individualized therapy, the TDM based dose optimization approach alone is not powerful enough to enable precision dosing for individual patients. The traditional TDM approach is associated with several limitations including the need for steady-state for sample collection, leading to delayed and suboptimal attainment of the PK/PD targets (Wicha et al., 2021). To address this, statistical and mathematical techniques are being applied using TDM results in dose optimization with the use of dosing software (Chai et al., 2020). Model-informed precision dosing (MIPD) is a mathematical framework that integrates different sources of information to streamline the TDM process and maximize the success of antibacterial therapy. To provide precise dosing, many other factors and parameters must be taken into consideration. This is where PK/PD models play a crucial role where the models provide information on drug exposure and effect either efficacy or toxicity (Wakefield et al., 1999). Furthermore, the incorporation of population PK and PK/PD models using the Bayesian approach and dosing simulations (Monte Carlo simulation) are utilized in MIPD. For some antibiotics, MIPD has been successfully developed (Roggeveen et al., 2020) and even implemented, for example, vancomycin (Frymoyer et al., 2020). As there is a demand for MIPD, it is not surprising that software tools have been developed to meet this need. Recent reviews (Kantasiripitak et al., 2020; Abdulla et al., 2021) reported there are around 10 MIPD software tools available, as shown in Table 3 below.

**TABLE 3:** Characteristics of model-informed precision dosing (MIPD) tools.

MIPD tool	Country	Mathematical software	Performance^#^ (%) > 75%
Autokinetics	Netherlands	NONMEM^®^, R^®^	X
Bestdose	United States	—	X
DoseMeRx	United States	GNU scientific library	✓
ID-ODS	United States	Matlab^®^	X
InsightRX nova	United States	NONMEM^®^	✓
MwPharm++	Netherlands/Czech Republic	—	✓
NextDose	New Zealand	—	X
PrecisePK	United States	—	✓
TDMx	Germany	NONMEM^®^	X
Tucuxi	Switzerland	NONMEM^®^	X

Adapted from Abdulla et al., 2021; Kantasiripitak et al., 2020. NONMEM: non-linear mixed effects model.

^#^Criteria include user-friendliness and utilization, user support, computational aspects, population models, quality and validation, output generation, privacy, data security, and costs (Kantasiripitak et al., 2020).

Although there are tools already available, there are still gaps in addressing unmet needs. As shown in Table 3, the performance of MIPD tools is based on 8 criteria: user-friendliness and utilization, user support, population models, quality and validation, output generation, privacy, data security and costs. Only four tools had a performance of more than 75%, with the highest scoring 83%. Thus, there is still room for improvement, including in aspects of validation and prospective evidence. Another crucial aspect of MIPD is continuous learning (Hughes et al., 2021). This involves the continuous update of the models and hence software tools based on data availability.

Notably, two MIPD tools have demonstrated cost-effectiveness, while for other products, trials are ongoing (Kantasiripitak et al., 2020). A study in 2018 by Tong et al. reported that the costs associated with pneumonia in the United States from 2008 to 2014 remain substantial and is a burden on the US healthcare system (Tong et al., 2018). Collectively, MIPD tools could potentially also save hospitalization costs in addition to saving lives.

## Future Directions

Applying and utilizing antimicrobial PK/PD models are crucial in optimizing antimicrobial therapy. Nonetheless, in the fight against antimicrobial resistance, novel strategies integrating mechanistic data from systems biology with antimicrobial PK/PD are warranted. As high-throughput data become more widely available and the demand for model-informed precision dosing (MIPD) increases, especially for narrow therapeutic window antibiotics, the need for QSP will also be more evident. MIPD will continue to evolve, thus requiring more information on biomarkers and mechanistic data. These can be obtained and provided from systems-based models, including QSP models. More studies investigating host-pathogen interactions and identifying biomarkers are crucial to further inform the models to enable optimization of antimicrobial therapy and precision dosing. As MIPD involves pharmacometrics, software and training costs must also be considered before they can be applied. Integration of MIPD tools with electronic health records must also be seamless for wider implementation. Lastly, cost-effectiveness studies of MIPD tools are scarce and therefore warranted. With the advent and availability of cost-effective, user-friendly and validated MIPD tools, clinicians will be able to further optimize antimicrobial therapy for their patients and thus health outcomes.

## References

[B1] Ab Rahman A. F. Ahmed Abdelrahim H. E. Mohamed Ibrahim M. I. (2013). A Survey of Therapeutic Drug Monitoring Services in Malaysia. Saudi Pharm. J. 21 (1), 19–24. 10.1016/j.jsps.2012.01.002 23960816 PMC3744941

[B2] Abdul Rahim N. Zhu Y. Cheah S.-E. Johnson M. D. Yu H. H. Sidjabat H. E. (2021). Synergy of the Polymyxin-Chloramphenicol Combination against New Delhi Metallo-β-Lactamase-Producing *Klebsiella pneumoniae* Is Predominately Driven by Chloramphenicol. ACS Infect. Dis. 7, 1584–1595. 10.1021/acsinfecdis.0c00661 33834753

[B3] Abdulla A. Edwina A. E. Flint R. B. Allegaert K. Wildschut E. D. Koch B. C. P. (2021). Model-Informed Precision Dosing of Antibiotics in Pediatric Patients: A Narrative Review. Front. Pediatr. 9, 624639. 10.3389/fped.2021.624639 33708753 PMC7940353

[B4] Aminov R. (2017). History of Antimicrobial Drug Discovery: Major Classes and Health Impact. Biochem. Pharmacol. 133, 4–19. 10.1016/j.bcp.2016.10.001 27720719

[B5] Appaneal H. J. Shireman T. I. Lopes V. V. Mor V. Dosa D. M. LaPlante K. L. (2021). Poor Clinical Outcomes Associated with Suboptimal Antibiotic Treatment Among Older Long-Term Care Facility Residents with Urinary Tract Infection: a Retrospective Cohort Study. BMC Geriatr. 21 (1), 1–3. 10.1186/s12877-021-02378-5 34301192 PMC8299613

[B6] Asín-Prieto E. Rodríguez-Gascón A. Isla A. (2015). Applications of the Pharmacokinetic/pharmacodynamic (PK/PD) Analysis of Antimicrobial Agents. J. Infect. Chemother. 21 (5), 319–329. 10.1016/j.jiac.2015.02.001 25737147

[B7] Balouiri M. Sadiki M. Ibnsouda S. K. (2016). Methods for *In Vitro* Evaluating Antimicrobial Activity: A Review. J. Pharm. Anal. 6 (2), 71–79. 10.1016/j.jpha.2015.11.005 29403965 PMC5762448

[B8] Cai Y. Yi J. Zhou C. Shen X. (2012). Pharmacogenetic Study of Drug-Metabolising Enzyme Polymorphisms on the Risk of Anti-tuberculosis Drug-Induced Liver Injury: a Meta-Analysis. PLoS One 7 (10), e47769. 10.1371/journal.pone.0047769 23082213 PMC3474796

[B9] Campbell J. H. O'Donoghue P. Campbell A. G. Schwientek P. Sczyrba A. Woyke T. (2013). UGA Is an Additional glycine Codon in Uncultured SR1 Bacteria from the Human Microbiota. Proc. Natl. Acad. Sci. U. S. A. 110, 5540–5545. 10.1073/pnas.1303090110 23509275 PMC3619370

[B10] Chai M. G. Cotta M. O. Abdul-Aziz M. H. Roberts J. A. (2020). What Are the Current Approaches to Optimising Antimicrobial Dosing in the Intensive Care Unit? Pharmaceutics 12 (7), 638. 10.3390/pharmaceutics12070638 PMC7407796 32645953

[B97] Cheng M. Cao L. Ning K. (2019). Microbiome Big-Data Mining and Applications Using Single-Cell Technologies and Metagenomics Approaches Toward Precision Medicine. Front. Genet., 972. 10.3389/fgene.2019.00972 31649735 PMC6794611

[B11] Chernov V. M. Chernova O. A. Mouzykantov A. A. Lopukhov L. L. Aminov R. I. (2019). Omics of Antimicrobials and Antimicrobial Resistance. Expert Opin. Drug Discov. 14 (5), 455–468. 10.1080/17460441.2019.1588880 30884978

[B12] Clinical & Laboratory Standards Institute (CLSI) (2022). CLSI Breakpoints. Available at: https://clsi.org/standards/products/free-resources/access-our-free-resources/ (Accessed 03 04, 2022).

[B13] Cohen A. Bont L. Engelhard D. Moore E. Fernández D. Kreisberg-Greenblatt R. (2015). A Multifaceted 'omics' Approach for Addressing the Challenge of Antimicrobial Resistance. Future Microbiol. 10 (3), 365–376. 10.2217/fmb.14.127 25812460

[B14] Craig W. A. (2003). Basic Pharmacodynamics of Antibacterials with Clinical Applications to the Use of Beta-Lactams, Glycopeptides, and Linezolid. Infect. Dis. Clin. North Am. 17 (3), 479–501. 10.1016/S0891-5520(03)00065-5 14711073

[B15] Czock D. Keller F. (2007). Mechanism-based Pharmacokinetic-Pharmacodynamic Modeling of Antimicrobial Drug Effects. J. Pharmacokinet. Pharmacodyn. 34 (6), 727–751. 10.1007/s10928-007-9069-x 17906920

[B16] Danhof M. de Lange E. C. Della Pasqua O. E. Ploeger B. A. Voskuyl R. A. (2008). Mechanism-based Pharmacokinetic-Pharmacodynamic (PK-PD) Modeling in Translational Drug Research. Trends Pharmacol. Sci. 29 (4), 186–191. 10.1016/j.tips.2008.01.007 18353445

[B17] de Araujo B. V. Diniz A. Palma E. C. Buffé C. Dalla Costa T. (2011). PK-PD Modeling of β-lactam Antibiotics: *In Vitro* or *In Vivo* Models? J. Antibiot. (Tokyo) 64 (6), 439–446. 10.1038/ja.2011.29 21505469

[B18] Derbalah A. Al-Sallami H. Hasegawa C. Gulati A. Duffull S. B. (2022). A Framework for Simplification of Quantitative Systems Pharmacology Models in Clinical Pharmacology. Br. J. Clin. Pharmacol. 88 (4), 1430–1440. 10.1111/bcp.14451 32621550

[B19] Dinos G. P. Athanassopoulos C. M. Missiri D. A. Giannopoulou P. C. Vlachogiannis I. A. Papadopoulos G. E. (2016). Chloramphenicol Derivatives as Antibacterial and Anticancer Agents: Historic Problems and Current Solutions. Antibiot. (Basel) 5. 10.3390/antibiotics5020020 PMC4929435 27271676

[B20] Dos Santos B. S. Da Silva L. C. da Silva T. D. Rodrigues J. F. Grisotto M. A. Correia M. T. (2016). Application of Omics Technologies for Evaluation of Antibacterial Mechanisms of Action of Plant-Derived Products. Front. Microbiol. 7, 1466. 10.3389/fmicb.2016.01466 27729901 PMC5037136

[B21] dos Santos K. V. Diniz C. G. de Castro Veloso L. de Andrade H. M. da Silva Giusta M. da Fonseca Pires S. (2010). Proteomic Analysis of *Escherichia coli* with Experimentally Induced Resistance to Piperacillin/tazobactam. Res. Microbiol. 161 (4), 268–275. 10.1016/j.resmic.2010.03.006 20381611

[B22] Dupont C. L. Rusch D. B. Yooseph S. Lombardo M. J. Richter R. A. Valas R. (2012). Genomic Insights to SAR86, an Abundant and Uncultivated Marine Bacterial Lineage. ISME J. 6 (6), 1186–1199. 10.1038/ismej.2011.189 22170421 PMC3358033

[B23] Dwyer D. J. Collins J. J. Walker G. C. (2015). Unraveling the Physiological Complexities of Antibiotic Lethality. Annu. Rev. Pharmacol. Toxicol. 55, 313–332. 10.1146/annurev-pharmtox-010814-124712 25251995

[B24] Ejim L. Farha M. A. Falconer S. B. Wildenhain J. Coombes B. K. Tyers M. (2011). Combinations of Antibiotics and Nonantibiotic Drugs Enhance Antimicrobial Efficacy. Nat. Chem. Biol. 7 (6), 348–350. 10.1038/nchembio.559 21516114

[B25] Erickson K. E. Otoupal P. B. Chatterjee A. (2017). Transcriptome-level Signatures in Gene Expression and Gene Expression Variability during Bacterial Adaptive Evolution. Msphere 2 (1), e00009–17. 10.1128/mSphere.00009-17 28217741 PMC5311112

[B26] European Committee on Antimicrobial Susceptibility Testing (EUCAST) (2022). Clinical Breakpoints - Breakpoints and Guidance. Available at: https://eucast.org/clinical_breakpoints/ (Accessed 03 04, 2022).

[B27] Fondi M. Liò P. (2015). Multi -omics and Metabolic Modelling Pipelines: Challenges and Tools for Systems Microbiology. Microbiol. Res. 171, 52–64. 10.1016/j.micres.2015.01.003 25644953

[B28] Frymoyer A. Schwenk H. T. Zorn Y. Bio L. Moss J. D. Chasmawala B. (2020). Model-Informed Precision Dosing of Vancomycin in Hospitalized Children: Implementation and Adoption at an Academic Children's Hospital. Front. Pharmacol. 11, 551. 10.3389/fphar.2020.00551 32411000 PMC7201037

[B29] Garcia E. Ly N. Diep J. K. Rao G. G. (2021). Moving from Point‐Based Analysis to Systems‐Based Modeling: Integration of Knowledge to Address Antimicrobial Resistance against MDR Bacteria. Clin Pharma Ther. 110 (5), 1196–1206. 10.1002/cpt.2219 33624298

[B30] Garza D. R. Dutilh B. E. (2015). From Cultured to Uncultured Genome Sequences: Metagenomics and Modeling Microbial Ecosystems. Cell Mol. Life Sci. 72 (22), 4287–4308. 10.1007/s00018-015-2004-1 26254872 PMC4611022

[B31] Groussin M. Poyet M. Sistiaga A. Kearney S. M. Moniz K. Noel M. (2021). Elevated Rates of Horizontal Gene Transfer in the Industrialized Human Microbiome. Cell 184 (8), 2053–e18. 10.1016/j.cell.2021.02.052 33794144

[B32] Hao T. Wu D. Zhao L. Wang Q. Wang E. Sun J. (2018). The Genome-Scale Integrated Networks in Microorganisms. Front. Microbiol. 9, 296. 10.3389/fmicb.2018.00296 29527198 PMC5829631

[B33] He S. Leanse L. G. Feng Y. (2021). Artificial Intelligence and Machine Learning Assisted Drug Delivery for Effective Treatment of Infectious Diseases. Adv. Drug Deliv. Rev. 178, 113922. 10.1016/j.addr.2021.113922 34461198

[B34] Hoo G. S. R. Liew Y. X. Kwa A. L. (2017). Optimisation of Antimicrobial Dosing Based on Pharmacokinetic and Pharmacodynamic Principles. Indian J. Med. Microbiol. 35 (3), 340–346. 10.4103/ijmm.IJMM_17_278 29063877

[B35] Hughes J. H. Tong D. M. H. Lucas S. S. Faldasz J. D. Goswami S. Keizer R. J. (2021). Continuous Learning in Model-Informed Precision Dosing: A Case Study in Pediatric Dosing of Vancomycin. Clin. Pharmacol. Ther. 109 (1), 233–242. 10.1002/cpt.2088 33068298 PMC7839485

[B36] Jorda A. Zeitlinger M. (2020). Preclinical Pharmacokinetic/Pharmacodynamic Studies and Clinical Trials in the Drug Development Process of EMA-Approved Antibacterial Agents: A Review. Clin. Pharmacokinet. 59 (9), 1071–1084. 10.1007/s40262-020-00892-0 32356105 PMC7467913

[B94] Kantasiripitak W. Van Daele R. Gijsen M. Ferrante M. Spriet I. Dreesen E. (2020). Software Tools for Model-Informed Precision Dosing: How Well Do They Satisfy the Needs?. Front. Pharmacol. 11, 620. 10.3389/fphar.2020.00620 32457619 PMC7224248

[B37] Khan D. D. Lagerbäck P. Cao S. Lustig U. Nielsen E. I. Cars O. (2015). A Mechanism-Based Pharmacokinetic/pharmacodynamic Model Allows Prediction of Antibiotic Killing from MIC Values for WT and Mutants. J. Antimicrob. Chemother. 70 (11), 3051–3060. 10.1093/jac/dkv233 26349518

[B38] Kim S. H. Kim S. H. Bahn J. W. Kim Y. K. Chang Y. S. Shin E. S. (2009). Genetic Polymorphisms of Drug-Metabolizing Enzymes and Anti-TB Drug-Induced Hepatitis. Pharmacogenomics 10 (11), 1767–1779. 10.2217/pgs.09.100 19891553

[B39] Kiratisin P. Chongthaleong A. Tan T. Y. Lagamayo E. Roberts S. Garcia J. (2012). Comparative *In Vitro* Activity of Carbapenems against Major Gram-Negative Pathogens: Results of Asia-Pacific Surveillance from the COMPACT II Study. Int. J. Antimicrob. Agents 39 (4), 311–316. 10.1016/j.ijantimicag.2012.01.002 22386743

[B40] Knight-Schrijver V. R. Chelliah V. Cucurull-Sanchez L. Le Novère N. (2016). The Promises of Quantitative Systems Pharmacology Modelling for Drug Development. Comput. Struct. Biotechnol. J. 14, 363–370. 10.1016/j.csbj.2016.09.002 27761201 PMC5064996

[B41] Kowalska-Krochmal B. Dudek-Wicher R. (2021). The Minimum Inhibitory Concentration of Antibiotics: Methods, Interpretation, Clinical Relevance. Pathogens 10 (2), 165. 10.3390/pathogens10020165 33557078 PMC7913839

[B42] Lai S.-J. Tu I.-F. Wu W.-L. Yang J.-T. Luk L. Y. P. Lai M.-C. (2017). Site-specific His/Asp Phosphoproteomic Analysis of Prokaryotes Reveals Putative Targets for Drug Resistance. BMC Microbiol. 17 (1), 1–10. 10.1186/s12866-017-1034-2 28545444 PMC5445275

[B43] Landersdorfer C. B. Nguyen T.-H. Lieu L. T. Nguyen G. Bischof R. J. Meeusen E. N. (2017). Substantial Targeting Advantage Achieved by Pulmonary Administration of Colistin Methanesulfonate in a Large-Animal Model. Antimicrob. Agents Chemother. 61 (1). 10.1128/aac.01934-16 PMC5192148 27821445

[B44] Leblebicioglu H. Cakir N. Cakir N. Celen M. Kurt H. Baris H. (2012). Comparative Activity of Carbapenem Testing (The COMPACT Study) in Turkey. BMC Infect. Dis. 12 (1), 1–8. 10.1186/1471-2334-12-42 22340940 PMC3298475

[B45] Lin Y.-W. Zhou Q. T. Han M.-L. Onufrak N. J. Chen K. Wang J. (2018). Mechanism-Based Pharmacokinetic/Pharmacodynamic Modeling of Aerosolized Colistin in a Mouse Lung Infection Model. Antimicrob. Agen. Chemother. 62 (3), e01965–e01917. 10.1128/AAC.01965-17 PMC5826166 29263069

[B46] Llanos-Paez C. C. Hennig S. Staatz C. E. (2017). Population Pharmacokinetic Modelling, Monte Carlo Simulation and Semi-mechanistic Pharmacodynamic Modelling as Tools to Personalize Gentamicin Therapy. J. Antimicrob. Chemother. 72 (3), 639–667. 10.1093/jac/dkw461 28062683

[B47] Magill S. S. O'Leary E. Ray S. M. Kainer M. A. Evans C. Bamberg W. M. (2021). Assessment of the Appropriateness of Antimicrobial Use in US Hospitals. JAMA Netw. Open 4 (3), e212007. 10.1001/jamanetworkopen.2021.2007 33734417 PMC7974639

[B48] McAleenan A. Ambrose P. G. Bhavnani S. M. Drusano G. L. Hope W. W. Mouton J. W. (2020). Methodological Features of Clinical Pharmacokinetic-Pharmacodynamic Studies of Antibacterials and Antifungals: a Systematic Review. J. Antimicrob. Chemother. 75 (6), 1374–1389. 10.1093/jac/dkaa005 32083674

[B49] Morales-Alvarez M. C. (2020). Nephrotoxicity of Antimicrobials and Antibiotics. Adv. Chronic Kidney Dis. 27 (1), 31–37. 10.1053/j.ackd.2019.08.001 32146999

[B50] Mouton J. W. Muller A. E. Canton R. Giske C. G. Kahlmeter G. Turnidge J. (2018). MIC-based Dose Adjustment: Facts and Fables. J. Antimicrob. Chemother. 73 (3), 564–568. 10.1093/jac/dkx427 29216348

[B51] Musante C. Ramanujan S. Schmidt B. Ghobrial O. Lu J. Heatherington A. (2017). Quantitative Systems Pharmacology: A Case for Disease Models. Clin. Pharmacol. Ther. 101, 24–27. 10.1002/cpt.528 27709613 PMC5217891

[B52] Muyderman H. Sims N. R. Tanaka M. Fuku N. Raghupathi R. Thyagarajan D. (2012). The Mitochondrial T1095C Mutation Increases Gentamicin-Mediated Apoptosis. Mitochondrion 12 (4), 465–471. 10.1016/j.mito.2012.06.006 22735573

[B53] Nielsen E. I. Friberg L. E. (2013). Pharmacokinetic-pharmacodynamic Modeling of Antibacterial Drugs. Pharmacol. Rev. 65 (3), 1053–1090. 10.1124/pr.111.005769 23803529

[B54] Nielsen E. I. Viberg A. Löwdin E. Cars O. Karlsson M. O. Sandström M. (2007). Semimechanistic Pharmacokinetic/pharmacodynamic Model for Assessment of Activity of Antibacterial Agents from Time-Kill Curve Experiments. Antimicrob. Agents Chemother. 51 (1), 128–136. 10.1128/aac.00604-06 17060524 PMC1797646

[B55] Nobu M. K. Narihiro T. Rinke C. Kamagata Y. Tringe S. G. Woyke T. (2015). Microbial Dark Matter Ecogenomics Reveals Complex Synergistic Networks in a Methanogenic Bioreactor. ISME J. 9 (8), 1710–1722. 10.1038/ismej.2014.256 25615435 PMC4511927

[B92] Ortwine J. K. Kaye K. S. Li J. Pogue J. M. (2015). Colistin: Understanding and Applying Recent Pharmacokinetic Advances Pharmacotherapy. J. Hum. Pharmacol. Drug Ther. 35 (1), 11–16. 10.1002/phar.1484 25187500

[B56] Pacheu-Grau D. Gómez-Durán A. López-Pérez M. J. Montoya J. Ruiz-Pesini E. (2010). Mitochondrial Pharmacogenomics: Barcode for Antibiotic Therapy. Drug Discov. Today 15 (1-2), 33–39. 10.1016/j.drudis.2009.10.008 19883791

[B57] Pai M. P. Bearden D. T. (2007). Antimicrobial Dosing Considerations in Obese Adult Patients. Pharmacotherapy 27 (8), 1081–1091. 10.1592/phco.27.8.1081 17655508

[B58] Palm N. W. de Zoete M. R. Cullen T. W. Barry N. A. Stefanowski J. Hao L. (2014). Immunoglobulin A Coating Identifies Colitogenic Bacteria in Inflammatory Bowel Disease. Cell 158, 1000–1010. 10.1016/j.cell.2014.08.006 25171403 PMC4174347

[B59] Peck R. W. (2021). Precision Dosing: An Industry Perspective. Clin. Pharmacol. Ther. 109, 47–50. 10.1002/cpt.2064 33107023 PMC7820949

[B60] Pérez-Pitarch A. Ferriols-Lisart R. Aguilar G. Ezquer-Garín C. Belda F. J. Guglieri-López B. (2018). Dosing of Caspofungin Based on a Pharmacokinetic/pharmacodynamic Index for the Treatment of Invasive Fungal Infections in Critically Ill Patients on Continuous Venovenous Haemodiafiltration. Int. J. Antimicrob. Agents 51 (1), 115–121. 10.1016/j.ijantimicag.2017.05.013 28666752

[B61] Pichardo-Almarza C. Diaz-Zuccarini V. (2016). From PK/PD to QSP: Understanding the Dynamic Effect of Cholesterol-Lowering Drugs on Atherosclerosis Progression and Stratified Medicine. Curr. Pharm. Des. 22, 6903–6910. 10.2174/1381612822666160905095402 27592718 PMC5403958

[B62] Pulido M. R. García-Quintanilla M. Gil-Marqués M. L. McConnell M. J. (2016). Identifying Targets for Antibiotic Development Using Omics Technologies. Drug Discov. Today 21 (3), 465–472. 10.1016/j.drudis.2015.11.014 26691873

[B63] Rawson T. M. Wilson R. C. O’Hare D. Herrero P. Kambugu A. Lamorde M. (2021). Optimizing Antimicrobial Use: Challenges, Advances and Opportunities. Nat. Rev. Microbiol. 19 (12), 747–758. 10.1038/s41579-021-00578-9 34158654

[B64] Reddy P. J. Ray S. Sathe G. J. Prasad T. S. K. Rapole S. Panda D. (2015). Proteomics Analyses ofBacillus Subtilisafter Treatment with Plumbagin, a Plant-Derived Naphthoquinone. Omics a J. Integr. Biol. 19 (1), 12–23. 10.1089/omi.2014.0099 PMC4281856 25562197

[B65] Ribba B. Grimm H. P. Agoram B. Davies M. R. Gadkar K. Niederer S. (2017). Methodologies for Quantitative Systems Pharmacology (QSP) Models: Design and Estimation. CPT Pharmacometrics Syst. Pharmacol. 6, 496–498. 10.1002/psp4.12206 28585415 PMC5572127

[B66] Roberts J. A. Paul S. K. Akova M. Bassetti M. De Waele J. J. Dimopoulos G. (2014). DALI: Defining Antibiotic Levels in Intensive Care Unit Patients: Are Current β-lactam Antibiotic Doses Sufficient for Critically Ill Patients? Clin. Infect. Dis. 58, 1072–1083. 10.1093/cid/ciu027 24429437

[B67] Rodríguez-Gascón A. Solinís M. Á. Isla A. (2021). The Role of PK/PD Analysis in the Development and Evaluation of Antimicrobials. Pharmaceutics 13 (6), 833. 10.3390/pharmaceutics13060833 34205113 PMC8230268

[B95] Roemer T. Boone C. (2013). Systems-Level Antimicrobial Drug and Drug Synergy Discovery. Nat. Chem. Biol. 9 (4), 222–231. 10.1038/nchembio.1205 23508188

[B68] Roggeveen L. F. Guo T. Driessen R. H. Fleuren L. M. Thoral P. van der Voort P. H. J. (2020). Right Dose, Right Now: Development of AutoKinetics for Real Time Model Informed Precision Antibiotic Dosing Decision Support at the Bedside of Critically Ill Patients. Front. Pharmacol. 11, 646. 10.3389/fphar.2020.00646 32499697 PMC7243359

[B69] Rydzanicz M. Wróbel M. Pollak A. Gawecki W. Brauze D. Kostrzewska-Poczekaj M. (2010). Mutation Analysis of Mitochondrial 12S rRNA Gene in Polish Patients with Non-syndromic and Aminoglycoside-Induced Hearing Loss. Biochem. Biophys. Res. Commun. 395 (1), 116–121. 10.1016/j.bbrc.2010.03.149 20353758

[B70] Schelli K. Zhong F. Zhu J. (2017). Comparative Metabolomics Revealing *Staphylococcus aureus* Metabolic Response to Different Antibiotics. Microb. Biotechnol. 10 (6), 1764–1774. 10.1111/1751-7915.12839 28815967 PMC5658637

[B96] Scheffler R. J. Colmer S. Tynan H. Demain A. L. Gullo V. P. (2013). Antimicrobials, Drug Discovery, and Genome Mining. Appl. Microbiol. Biotechnol. 97 (3), 969–978. 10.1007/s00253-012-4609-8 23233204

[B71] Schmidt S. Barbour A. Sahre M. Rand K. H. Derendorf H. (2008). PK/PD: New Insights for Antibacterial and Antiviral Applications. Curr. Opin. Pharmacol. 8 (5), 549–556. 10.1016/j.coph.2008.06.010 18625339

[B72] Shen F. Tang X. Wang Y. Yang Z. Shi X. Wang C. (2015). Phenotype and Expression Profile Analysis of *Staphylococcus aureus* Biofilms and Planktonic Cells in Response to Licochalcone A. Appl. Microbiol. Biotechnol. 99 (1), 359–373. 10.1007/s00253-014-6076-x 25256617

[B73] Shin S. J. Kang S. G. Nabin R. Kang M. L. Yoo H. S. (2005). Evaluation of the Antimicrobial Activity of Florfenicol against Bacteria Isolated from Bovine and Porcine Respiratory Disease. Vet. Microbiol. 106, 73–77. 10.1016/j.vetmic.2004.11.015 15737475

[B74] Singh R. Sripada L. Singh R. (2014). Side Effects of Antibiotics during Bacterial Infection: Mitochondria, the Main Target in Host Cell. Mitochondrion 16, 50–54. 10.1016/j.mito.2013.10.005 24246912

[B75] Sorger P. K. Allerheiligen S. R. B. Abernethy D. R. Altmann R. B. Brouwer K. L. R. Califano A. (2011). “Quantitative and Systems Pharmacology in the Post-Genomic Era: New Approaches to Discovering Drugs and Understanding Therapeutic Mechanisms (White Paper),” in An NIH White Paper by the QSP Workshop Group (Rockville, MD, USA: Bethesda), 48.

[B76] Tong S. Amand C. Kieffer A. Kyaw M. H. (2018). Trends in Healthcare Utilization and Costs Associated with Pneumonia in the United States during 2008-2014. BMC Health Serv. Res. 18, 715. 10.1186/s12913-018-3529-4 30217156 PMC6137867

[B77] Trang M. Dudley M. N. Bhavnani S. M. (2017). Use of Monte Carlo Simulation and Considerations for PK-PD Targets to Support Antibacterial Dose Selection. Curr. Opin. Pharmacol. 36, 107–113. 10.1016/j.coph.2017.09.009 29128853

[B78] Tuntland T. Ethell B. Kosaka T. Blasco F. Zang R. X. Jain M. (2014). Implementation of Pharmacokinetic and Pharmacodynamic Strategies in Early Research Phases of Drug Discovery and Development at Novartis Institute of Biomedical Research. Front. Pharmacol. 5, 174. 10.3389/fphar.2014.00174 25120485 PMC4112793

[B79] Valenza G. Seifert H. Decker-Burgard S. Laeuffer J. Morrissey I. Mutters R. (2012). Comparative Activity of Carbapenem Testing (COMPACT) Study in Germany. Int. J. Antimicrob. Agents 39 (3), 255–258. 10.1016/j.ijantimicag.2011.10.015 22230334

[B80] van der Graaf P. H. Benson N. (2011). Systems Pharmacology: Bridging Systems Biology and Pharmacokinetics-Pharmacodynamics (PKPD) in Drug Discovery and Development. Pharm. Res. 28, 1460–1464. 10.1007/s11095-011-0467-9 21560018

[B93] Velkov T. Bergen P. J. Lora-Tamayo J. Landersdorfer C. B. Li J. (2013). PK/PD Models in Antibacterial Development. Curr. Opin. Microbiol. 16 (5), 573–579. 10.1016/j.mib.2013.06.010 23871724 PMC3834155

[B81] Wakefield J. Aarons L. Racine-Poon A. (1999). “The Bayesian Approach to Population Pharmacokinetic/pharmacodynamic Modeling,” in Case Studies in Bayesian Statistics. Lecture Notes in Statistics. Editor Gatsonis C. (New York, NY: Springer), 140. 10.1007/978-1-4612-1502-8_4

[B82] Wang Z. Gerstein M. Snyder M. (2009). RNA-seq: a Revolutionary Tool for Transcriptomics. Nat. Rev. Genet. 10 (1), 57–63. 10.1038/nrg2484 19015660 PMC2949280

[B83] Wei C. F. Shien J. H. Chang S. K. Chou C. C. (2016). Florfenicol as a Modulator Enhancing Antimicrobial Activity: Example Using Combination with Thiamphenicol against *Pasteurella Multocida* . Front. Microbiol. 7, 389. 10.3389/fmicb.2016.00389 27065961 PMC4811925

[B84] Wicha S. G. Märtson A. G. Nielsen E. I. Koch B. C. P. Friberg L. E. Alffenaar J. W. (2021). From Therapeutic Drug Monitoring to Model-Informed Precision Dosing for Antibiotics. Clin. Pharmacol. Ther. 109 (4), 928–941. 10.1002/cpt.2202 33565627

[B85] Woodhead J. L. Brock W. J. Roth S. E. Shoaf S. E. Brouwer K. L. Church R. (2017). Application of a Mechanistic Model to Evaluate Putative Mechanisms of Tolvaptan Drug-Induced Liver Injury and Identify Patient Susceptibility Factors. Toxicol. Sci. 155, 61–74. 10.1093/toxsci/kfw193 27655350 PMC5216653

[B86] Wozniak J. M. Mills R. H. Olson J. Caldera J. R. Sepich-Poore G. D. Carrillo-Terrazas M. (2020). Mortality Risk Profiling of *Staphylococcus aureus* Bacteremia by Multi-Omic Serum Analysis Reveals Early Predictive and Pathogenic Signatures. Cell 182 (5), 1311–e14. 10.1016/j.cell.2020.07.040 32888495 PMC7494005

[B87] Yadav R. Bulitta J. B. Wang J. Nation R. L. Landersdorfer C. B. (2017). Evaluation of Pharmacokinetic/Pharmacodynamic Model-Based Optimized Combination Regimens against Multidrug-Resistant *Pseudomonas aeruginosa* in a Murine Thigh Infection Model by Using Humanized Dosing Schemes. Antimicrob. Agents Chemother. 61, e01268–17. 10.1128/AAC.01268-17 PMC5700304 28993331

[B88] Yapa S. W. S. Li J. Patel K. Wilson J. W. Dooley M. J. George J. (2014). Pulmonary and Systemic Pharmacokinetics of Inhaled and Intravenous Colistin Methanesulfonate in Cystic Fibrosis Patients: Targeting Advantage of Inhalational Administration. Antimicrob. Agents Chemother. 58 (5), 2570–2579. 10.1128/AAC.01705-13 24550334 PMC3993267

[B89] Zhang A. N. Gaston J. M. Dai C. L. Zhao S. Poyet M. Groussin M. (2021). An Omics-Based Framework for Assessing the Health Risk of Antimicrobial Resistance Genes. Nat. Commun. 4765, 1–11. 10.1038/s41467-021-25096-3 PMC8346589 34362925

[B90] Zhao S. Iyengar R. (2012). Systems Pharmacology: Network Analysis to Identify Multiscale Mechanisms of Drug Action. Annu. Rev. Pharmacol. Toxicol. 52, 505–521. 10.1146/annurev-pharmtox-010611-134520 22235860 PMC3619403

[B91] Zhu Y. Czauderna T. Zhao J. Klapperstueck M. Maifiah M. H. M. Han M. L. (2018). Genome-scale Metabolic Modeling of Responses to Polymyxins in *Pseudomonas aeruginosa* . Gigascience 7, giy021. 10.1093/gigascience/giy021 PMC6333913 29688451

